# DINOv3-Driven Semantic Segmentation for Landslide Mapping in Mountainous Regions

**DOI:** 10.3390/s26020406

**Published:** 2026-01-08

**Authors:** Zhiyi Dou, Edore Akpokodje, Yuelin He, Yuxin Liu, Zixuan Ni, Chang’an Xu, Muhammad Aslam, Meng Tang

**Affiliations:** 1 School of Earth and Environmental Sciences, Faculty of Science, The University of Queensland, Brisbane, QLD 4072, Australia; zhiyi.dou@uq.net.au; 2 Department of Computer Science, Aberystwyth University, Penglais, Aberystwyth SY23 3DB, UK; eta@aber.ac.uk (E.A.); yuh22@aber.ac.uk (Y.H.); yuxinliu25@stu.pku.edu.cn (Y.L.); mua19@aber.ac.uk (M.A.); 3 Department of Computer Science, Peking University, Beijing 100871, China; 4 State Key Laboratory of Information Engineering in Surveying, Mapping and Remote Sensing, Wuhan University, Wuhan 430079, China; 2022106190059@whu.edu.cn; 5 Institute of Chemical Engineering, Guangdong Academy of Sciences, Guangzhou 510665, China; xuchangan@gdcri.com

**Keywords:** landslide segmentation, multi-source data integration, cross-sensor applicability, sensor processing, remote sensing

## Abstract

Landslide hazard assessment increasingly demands the joint analysis of heterogeneous remote sensing data; however, automating this process remains difficult due to the pronounced resolution and texture discrepancies existing between satellite and aerial sensors. To address these limitations, this study proposes a robust segmentation framework capable of extracting sensor-robust representations. The framework leverages a DINOv3 transformer encoder and exploits representations from multiple transformer layers to capture complementary visual information, ranging from fine-grained surface textures to global semantic contexts, overcoming the receptive field constraints of conventional CNNs. Experiments on the Longxi satellite dataset achieve a Dice coefficient of 0.96 and an IoU of 0.938, and experiments on the Longxi UAV dataset achieve a Dice coefficient of 0.965 and an IoU of 0.941. These results show consistent segmentation performance on both the Longxi satellite and UAV datasets, despite differences in spatial resolution and surface appearance between acquisition platforms.

## 1. Introduction

Under the dual effects of intensified global climate change and the expansion of human engineering activities, the problem of imbalanced stability of slope rock and soil mass has become increasingly prominent, and landslides have become one of the main geological hazards threatening the safety of mountainous areas [1]. Landslide refers to the natural phenomenon of soil or rock mass on a slope sliding down along a specific weak surface or zone under the action of gravity [2]. Its essence is the mechanical imbalance process where the shear stress on the sliding surface exceeds the shear strength [3]. The core characteristics of landslides can be clearly presented via three dimensions: the classification basis, the formation conditions, and the development process, and each feature is closely connected [4]. First, they can be divided into four core types according to their material composition and movement characteristics. Among them, rock landslides [5] often occur in areas with hard rock layers and are caused by the development of joint fissures or seismic damage to the integrity of rock layers; soil landslides [6] are common in areas covered by loose soil layers, and the main cause is the decrease in soil strength due to rainfall infiltration; mudstone landslides are formed by mixing soil and rock mixtures with water bodies, possessing the dual characteristics of sliding and flow [7]; and collapse manifests as a rapid drop of local rock and soil mass from the parent body of the slope, often occurring on steep slopes [8]. Second, the formation of landslides requires two basic conditions: terrain conditions, namely, the sliding space in front of the slope and the cutting surfaces on both sides [9]; and induced dynamics, including external factors such as rainfall, earthquakes, groundwater activity, and artificial slope cutting [10]. From the perspective of developmental laws, the occurrence of landslides is not sudden; it gradually progresses through four stages: creep deformation, rapid deformation, sliding, and stability. This process is closely related to the aforementioned material composition and formation conditions and constitutes the complete system of landslide characteristics.

### 1.1. Global Impact of Landslides

Landslides are one of the most widely distributed and long-lasting geological disasters in the world, causing shocking casualties and economic losses. According to the latest report by the United Nations International Strategy for Disaster Reduction in 2023, there are an average of over 4000 landslides worldwide each year, resulting in nearly 500,000 deaths between 2010 and 2020 alone. The average number of deaths per disaster far exceeds that of floods and earthquakes. In terms of economic losses, the global annual direct losses caused by landslides amount to about 10–20 billion US dollars. If combined with indirect costs such as transportation disruptions and ecological restoration, the total amount can reach 3–5 times the direct losses [11]. The dual drivers of climate change and human activities are continuously exacerbating the risk and intensity of landslide disasters. At the climate level, global warming has led to an increase in the frequency of extreme precipitation events of over 30%. High-intensity rainfall infiltrates rock and soil in a short period of time, causing a sharp drop in their shear strength. For example, the incidence of landslides during the summer monsoon season in the Himalayan region of India has increased by 45% compared to the end of the 20th century. At the same time, the accelerated melting of high mountain glaciers has caused glacial lake outburst floods, which have become a new trigger for landslides in high-altitude areas. At the level of human activities, the excessive reclamation, road excavation, and engineering construction in Venezuela’s Avila Mountains, China’s Jiuzhaigou Valley, an area of scenic and historic interest, and other regions have damaged the original stability of the mountains, reduced the trigger threshold of landslides, and increased the probability of disasters under similar precipitation conditions by 2–3 times [12]. From typical global cases, the destructive power of landslide disasters has distinct commonalities: in the Himalayan region of India, due to the combination of plate activity and monsoon rainfall, frequent landslides block transportation arteries more than 200 times a year, damaging about 12,000 hectares of farmland [13]. In 1999, the landslide of Venezuela’s Avila Mountain caused a huge flash flood due to rainstorm and structural damage to the mountain, resulting in more than 30,000 deaths and economic losses of USD 1.5 billion [12]. In the Jiuzhaigou Valley area, as shown in Table 1, 15% of the natural landscape in the core scenic area was damaged due to landslides induced by earthquakes and heavy rainfall, and the direct loss of tourism industry exceeded 2 billion yuan [14]. The global, sudden, and highly destructive nature of landslide disasters has become a core bottleneck restricting the sustainable development of mountainous areas. Traditional ground monitoring methods struggle to cover the vast and complex terrain of mountainous areas, and the demand for efficient and accurate remote sensing monitoring is becoming increasingly urgent. To overcome the drawbacks of low efficiency and strong subjectivity in remote sensing image interpretation, automated semantic segmentation technology based on deep learning can achieve rapid identification and range extraction of landslide areas, providing key technical support for disaster warning and emergency response. This has also become one of the core research directions in the field of landslide disaster monitoring.

### 1.2. Landslide Events in Research Area

**Figure 1:**
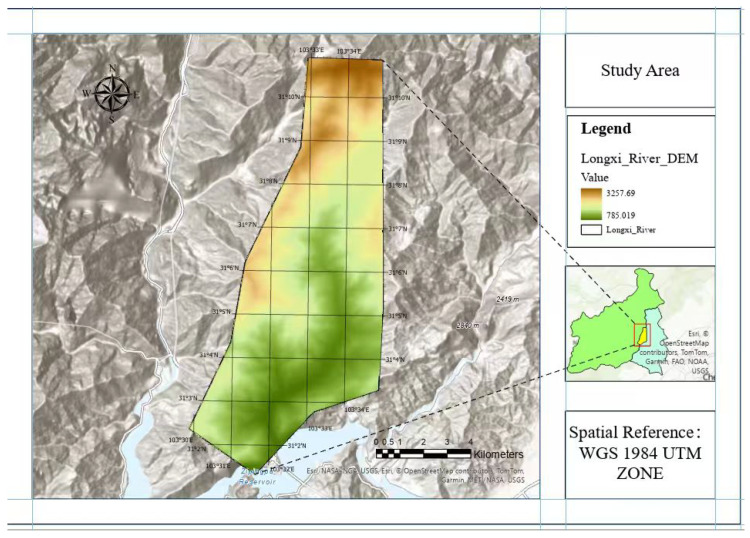
Digital elevation map (DEM) of the Longxi River Basin, China.

China’s Longxi River Basin is located in the eastern edge of the Qinghai Tibet Plateau, where landslide disasters are common. The vertical climate differentiation in the basin is obvious: the monsoon brings concentrated rainstorms, and freezing–thawing alternates frequently, providing the climate conditions conducive to producing landslides. In addition, the geological environment is fragile and the basin is located on the western edge of the Sichuan Basin, significantly affected by the activity of the Longmen Mountain fault zone [15]. After the Wenchuan earthquake, the regional geological environment underwent fundamental changes, providing a broad material basis for landslides. The widely distributed sandstone and mudstone and loose accumulation-bodies in the watershed have formed a large number of longitudinal slopes under the action of tectonic movements. The topography of the Longxi River Basin is shown in Figure 1.

This combination of engineering geological rock formations and slope structures has become a key internal factor in the development of landslides [16]. The Longxi River landslide exhibits the distinct characteristics of group occurrence and disaster chain, with disasters mostly concentrated during the flood season [17]. A single heavy rainfall process can trigger the synchronous activity of dozens of landslides. Landslides are mostly distributed along both sides of the river valley, and their sliding paths are controlled by the valley terrain, often resulting in short distance high-speed sliding. Some landslide debris directly flows into the river channel, forming dam bodies and blocking water flow. This type of landslide has strong concealment characteristics, and the early signs of deformation are often covered by dense vegetation. It often occurs in remote mountainous areas with less human activity, which brings great difficulty to warning of the disaster in advance [18]. Landslide disasters have had a systematic impact on the Longxi River Basin. At the ecological level, vegetation stripping and soil erosion caused by landslides have damaged the integrity of the valley ecosystem, leading to a large amount of sediment entering the river and causing water quality deterioration and riverbed uplift. At the economic level, disasters have damaged rural roads, irrigation systems, and power facilities within the watershed, causing multiple villages to temporarily lose contact with the outside world and severely impacting agricultural production. At the social level, frequent landslide threats have forced coastal residents to relocate, changing the traditional settlement pattern. At the same time, post-disaster reconstruction requires the long-term investment of a large amount of resources, applying sustained pressure to local finances [19].

### 1.3. Technologies for Landslide Investigation

Landslide investigation is a core technical means of revealing the causes of disasters and assessing risk levels. Here, a multidimensional investigation system combining ground investigation, geological exploration, and remote sensing technology has been formed. Ground investigation is the basic method, employing slope units. The aim is to verify disaster-prone terrain and geological structure conditions based on the development law of geological hazards and to delineate potential hazards. In terms of operation, combined with previous data, the method employs geological compasses and other tools to record parameters, review remote sensing hidden danger points, and form a database. Actual measurement information can verify other results [20].

Geological exploration focuses on the interior of slopes, and the aim is to reveal the location of sliding zones and the mechanical properties of rock and soil masses through geophysical exploration and drilling. In terms of operation, a high-density electrical method is used to invert material distribution, drilling is used to obtain core analysis of strata, and through profile design, data collection, and other steps, ground investigation provides the parameters for stability assessment.

Remote sensing technology [21] has established a key macro perspective for landslide investigation, building an integrated monitoring system of “sky–air–ground.” Its core advantage lies in accurately capturing high-value data through various specialized sensors, breaking through the spatial and temporal limitations and efficiency bottlenecks of traditional investigations at the space-based level. Optical remote sensing relies on high-resolution optical sensors carried by platforms such as high-resolution series and sentinel satellites. It not only has a wide coverage area and fast data acquisition time but can also clearly restore surface texture, vegetation coverage changes, and building damage details. By establishing standard resolution markers, accurately delineating landslide damage areas and impact boundaries, it provides efficient support for regional-scale hidden danger screening and can intuitively present the spatiotemporal evolution trajectory of landslides through multi-phase image comparison, greatly reducing the blindness associated with manual exploration. The role of this sensor is particularly prominent. InSAR technology utilizes satellite radar sensors to penetrate clouds and rain, without being limited by day and night cycles, and captures the slow deformation of millimeter-scale slopes. In the airborne platform, unmanned aerial vehicle LiDAR combined with laser sensors can penetrate the vegetation canopy, obtain high-density 3D point cloud data, accurately restore micro terrain deformation details, and synchronously collect multispectral data to enrich stability judgment basis. This multi-technology collaborative mode not only reduces the manpower investment and safety risks in remote mountainous area investigation but also improves the initial investigation efficiency, relying on comprehensive and accurate data and thus laying a solid foundation for subsequent work. The advantages and limitations of these investigation methods are summarized in Table 2.

### 1.4. Specific Challenges Faced by Current Research Methods

Although significant progress has been made in landslide investigation technology, existing methods still face multiple challenges in complex geological environments and high-precision requirements, among which the limitations of remote sensing technology are particularly prominent. The complexity of geography imposes comprehensive constraints on survey methods. In high-altitude mountainous areas and deep canyon regions, ground surveys struggle to reach steep slopes and isolated mountains, posing extremely high risks to personnel safety. Geological exploration is limited by terrain, and determining the layout of profiles and transporting instruments are challenging, making it difficult to achieve uniform coverage. For remote sensing technology, the obstruction of dense vegetation makes it impossible for optical images to capture subtle surface deformations, while InSAR technology is affected by terrain slopes and is prone to observation geometric errors in steep areas. In addition, the cloudy and foggy climate in mountainous areas often leads to missing remote sensing data, forming monitoring blind spots, and seasonal changes in ice and snow covered areas can interfere with the extraction of deformation information, reducing the accuracy of data interpretation. The scarcity of data and difficulty in labeling have become key bottlenecks that constrain technological upgrades. In remote disaster-prone areas, there is insufficient deployment of long-term monitoring stations and a lack of continuous meteorological, deformation, and other basic data, resulting in a lack of quantitative support for landslide cause analysis. For remote sensing technology, the lack of high-quality annotated datasets seriously constrains the training performance of intelligent recognition models. The complex morphology of landslides and the blurred boundaries among newly occuring landslides, the surrounding bare land, reactivated ancient landslide bodies, and vegetated areas make accurate delineation challenging. Meanwhile, there are significant differences in landslide characteristics among different regions, and models trained on single regional data have insufficient generalization ability. Building cross-regional datasets requires a lot of manpower and resources, making it difficult to achieve large-scale accumulation in the short term [3]. There are still technical barriers to the collaborative fusion of existing methods. The lack of unified standards for ground surveys and remote sensing interpretation results in difficulty in effectively integrating data. The lack of precise matching methods between point data from geological exploration and surface information from remote sensing makes it impossible to achieve three-dimensional modeling from the surface to the underground. These issues are particularly prominent in complex terrain areas, leading to multiple solutions in survey results and difficulty in forming consistent disaster assessment conclusions, which constrains the scientific and accurate decision-making of landslide prevention and control.

### 1.5. Deep Learning-Based Semantic Segmentation for Landslide Mapping

CNN-based semantic segmentation is widely used for landslide mapping from remote sensing imagery because it captures local textures and boundary cues. Representative architectures include U-Net and its variants, such as U-Net++, PSPNet, and DeepLabv3+. These models are efficient and stable to train, but their local receptive fields limit long-range dependency modeling and global geomorphological understanding. This limitation affects the delineation of complex and fragmented landslide boundaries under different terrain and illumination conditions. Moreover, landslide mapping often involves class imbalance and visual ambiguity, such as spectral similarity to bare soil or river deposits, which increases boundary uncertainty.

Transformer-based segmentation uses self-attention to capture global context and performs well on dense prediction tasks. Vision Transformer and hierarchical backbones such as Swin Transformer [22] are widely used, and frameworks such as SegFormer [23] and Mask2Former [24] improve multi-scale feature modeling and mask prediction. For landslide mapping, Transformer-based models can better capture structural patterns and multi-scale textures, which helps extract irregular landslide shapes. In addition, global attention exploits long-range dependencies and contextual constraints, which helps distinguish landslides from visually similar background regions. Nevertheless, these models often require more computation, which is challenging in geoscience applications where pixel-level annotations are limited and costly.

Despite these advances, two issues remain important for practical landslide mapping: capturing fine boundary details while preserving geomorphological context across scales, and maintaining stable performance under different sensors and acquisition conditions. To address these issues, this work uses a self-supervised DINOv3 encoder and leverages representations from multiple transformer layers to improve boundary delineation in mountainous regions.

## 2. Dataset

Landslide disasters are characterized by strong suddenness and complex spatial distribution, posing significant challenges for surface monitoring and hazard prevention. With the continuous advancement of remote sensing observation technology, applying deep learning to multi-source remote sensing imagery has become a feasible and efficient approach for automatic landslide identification and segmentation. This study aims to develop an end-to-end deep learning-based framework for landslide segmentation to address the challenges posed by complex terrains and significant variations in illumination conditions.

We used subsets of the CAS-Landslide multi-sensor dataset [25], developed by the Artificial Intelligence Group at the Institute of Mountain Hazards and Environment, Chinese Academy of Sciences (CAS). To ensure a consistent study area while enabling multi-sensor comparison, we selected two datasets from the Longxi River Basin: Longxi River (UAV) and Longxi River (SAT), which were used for model development and evaluation, as shown in Table 3. The Longxi River (UAV) dataset, collected by the Sichuan Geomatics Center, covers the period from March to May 2011 and includes 2504 UAV images with a spatial resolution of 0.5 m, contributing diverse samples under varying geomorphic and illumination conditions for model training. In addition, the Longxi River (SAT) dataset was provided by the China Centre for Resources Satellite Data and Application. The imagery was captured by the GF-1 satellite between March and December 2015, comprising 1769 satellite images with a spatial resolution of 0.5 m. Due to its extensive coverage and complex imaging conditions, the Longxi River (SAT) dataset is used as the satellite benchmark for in-domain training, validation, and testing. In parallel, the Longxi River (UAV) dataset is used under the same in-domain protocol. Together, the two datasets enable a controlled multi-platform evaluation within the same study area, allowing us to compare segmentation performance under different acquisition platforms and sensors conditions.

## 3. Methodology

### 3.1. Overall Framework

**Figure 2:**
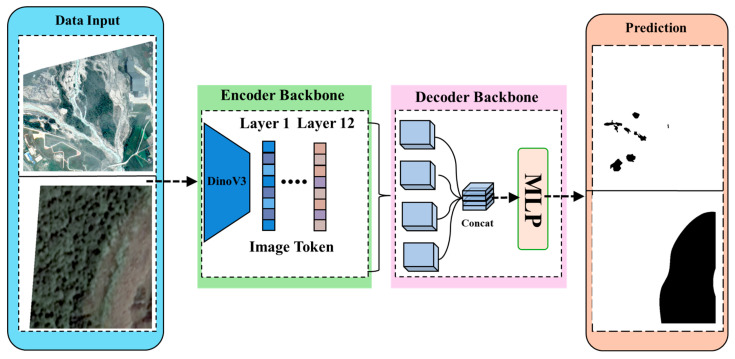
Overview of the proposed landslide segmentation framework. The architecture consists of a DINOv3 encoder backbone and a lightweight MLP decoder. Input images are processed to extract hierarchical visual tokens. Feature representations from multiple transformer layers (1 to 12) are concatenated to capture multi-scale context, which are then projected by the MLP to generate the final binary prediction mask.

The proposed framework is designed as an end-to-end system for landslide segmentation utilizing multi-source remote sensing imagery. As explicitly illustrated in Figure 2, the architecture comprises three integral components: (1) the preprocessing of UAV and satellite imagery; (2) hierarchical feature extraction via a DINOv3 encoder backbone; and (3) a lightweight MLP decoder responsible for generating the final segmentation map. During the preprocessing phase, input images undergo normalization and are partitioned into fixed-size patches to ensure data consistency and effectively capture local terrain variations. These patches are subsequently processed by the DINOv3 encoder. The encoder extracts visual tokens from various transformer layers: lower layers preserve fine-grained textures and edge details, whereas deeper layers encode abstract semantic information regarding landslide morphology. Leveraging these hierarchical features allows the model to simultaneously represent micro-scale surface changes and macro-scale structural patterns. Finally, the selected tokens are aggregated and forwarded to a streamlined MLP-based decoder, which projects the fused features into the output space to generate the pixel-level binary mask.

### 3.2. Our Proposed Segmentation Model

Our framework leverages the power of self-supervised learning to address the data scarcity challenge in landslide segmentation. Specifically, we employ a DINOv3 encoder, which learns robust visual representations from massive unlabeled data without relying on class-specific labels. Building on this strong foundation, the proposed architecture couples the encoder with a lightweight MLP decoder, as shown in Figure 2. In contrast to conventional approaches that rely solely on the final representation layer, our model aggregates feature tokens from all transformer layers. This multi-level integration is crucial for effectively capturing the multi-scale morphological characteristics inherent to landslides.

Given an input image $I\in R^{H\times W\times 3}$, the encoder first projects it into a sequence of initial embeddings:

(1) $$X_{0}=P\left(I\right),$$

where P(·) denotes the patch embedding operation. These tokens are iteratively refined through a stack of *L* Transformer layers:

(2) $$X_{\ell }=F_{\ell }\left(X_{\ell -1}\right),\;\ell =1,\ldots ,L,$$

where $F_{\ell }\left(\cdot \right)$ represents the *ℓ*-th Transformer block. This process yields a hierarchy of representations $\left\{X_{1},\ldots ,X_{L}\right\}$. To recover spatial structure, each token sequence is reshaped into a feature map:

(3) $$T_{\ell }=\mathrm{Reshape}\left(X_{\ell }\right),\;T_{\ell }\in R^{h\times w\times C},$$

Distinctively, shallow layers preserve high-frequency texture details, whereas deeper layers encode semantic morphological patterns. We aggregate these multi-scale features by concatenating them along the channel dimension:

(4) $$F=\mathrm{Concat}\left(T_{1},T_{2},\ldots ,T_{L}\right),\;F\in R^{h\times w\times (LC)}.$$

This fused tensor *F*, encapsulating both local nuances and global context, is then processed by a decoder function D(·). The decoder projects the features to the output space:

(5) $$\hat{Y}=\sigma \;\left(D(F)\right),\;\hat{Y}\in R^{H\times W},$$

where σ(·) denotes the sigmoid activation function, producing the final pixel-wise probability map for landslide segmentation.

### 3.3. Training Details and Evaluation Metrics

All models are trained on 256×256 patches derived from the multi-source imagery detailed in Section 2. To preclude spatial data leakage, we enforce an image-level partitioning strategy where patches originating from a single scene are exclusively assigned to the same subset. The dataset is split into training, validation, and testing sets with a ratio of 60%, 20%, and 20%, respectively. Prior to network ingestion, input samples are normalized and standardized based on channel-wise statistics calculated solely from the training partition. Furthermore, to bolster model robustness against environmental variability, the training pipeline incorporates comprehensive data augmentation techniques, ranging from random geometric flips and rotations to radiometric brightness and contrast jittering.

The model training is driven by the Adam optimizer, initialized with a learning rate of $1\times 10^{-3}$. To facilitate stable convergence and escape local minima, we employ a cosine annealing schedule that dynamically adjusts the learning rate throughout the training process. To strictly mitigate the risk of overfitting given the complexity of landslide features, a regularization strategy is implemented comprising a weight decay of $5\times 10^{-5}$ and a dropout rate of 0.3, specifically applied within the decoder module. Furthermore, gradient clipping with a maximum norm of 0.7 is utilized to stabilize backpropagation and prevent gradient explosion. The training procedure is monitored via an early stopping mechanism with a patience of 20 epochs based on validation loss performance. All computational experiments are executed on a single NVIDIA A100 GPU with 80 GB of memory, and the comprehensive set of hyperparameters is detailed in Table 4.

To effectively guide network optimization and mitigate the inherent class imbalance between scarce landslide pixels and the dominant background, we employ a composite loss function. This objective synergizes Binary Cross-Entropy (BCE) [26] loss with Dice loss [27]: the former ensures pixel-wise classification fidelity, while the latter optimizes geometric alignment, which is particularly crucial for delineating fragmented landslide instances.

Specifically, let Ω denote the image spatial domain. For each pixel i∈Ω, let $Y_{i}\in \left\{0,1\right\}$ represent the binary ground-truth label and ${\hat{Y}}_{i}\in \left[0,1\right]$ denote the predicted probability. The BCE loss is defined as follows:

(6) $$L_{\mathrm{BCE}}=-\frac{1}{\left|\Omega \right|}\sum_{i\in \Omega }\left(Y_{i}\log{\hat{Y}}_{i}+\left(1-Y_{i}\right)\log\left(1-{\hat{Y}}_{i}\right)\right).$$

To complement the pixel-level supervision, the Dice loss is incorporated to penalize the mismatch in regional overlap between predictions and ground truth:

(7) $$L_{\mathrm{Dice}}=1-\frac{2\sum_{i\in \Omega }Y_{i}{\hat{Y}}_{i}+\epsilon }{\sum_{i\in \Omega }Y_{i}+\sum_{i\in \Omega }{\hat{Y}}_{i}+\epsilon },$$

where ϵ is a smoothing factor introduced for numerical stability. The final training loss function L is formulated as an equal-weighted summation of these two terms to balance pixel accuracy with regional coherence:

(8) $$L=0.5\cdot L_{\mathrm{BCE}}+0.5\cdot L_{\mathrm{Dice}}.$$

Following the training phase, the model is deployed to generate predictions on the test set. For a given input, the network outputs a probability map $\hat{Y}$. To derive the final binary segmentation mask $\tilde{Y}$, we apply a standard threshold τ=0.5, where pixels with probabilities ${\hat{Y}}_{i}\geq \tau$ are classified as landslides.

To comprehensively assess the segmentation performance, we employ a set of standard metrics: Precision, Recall, Dice Coefficient, Intersection over Union (IoU), and Overall Accuracy (OA). While OA provides a general measure of correct predictions, it can be less sensitive to the minority class in imbalanced scenarios. In contrast, metrics like the Dice Coefficient and IoU specifically measure the overlap between the predicted landslide regions and the ground truth, offering more assessments of the model’s effectiveness in capturing the target hazard. The specific definitions are as follows:

(9) $$\mathrm{Precision}=\frac{\mathrm{TP}}{\mathrm{TP}+\mathrm{FP}},\;\mathrm{Recall}=\frac{\mathrm{TP}}{\mathrm{TP}+\mathrm{FN}},$$

(10) $$\mathrm{Dice}=\frac{2\;\mathrm{TP}}{2\;\mathrm{TP}+\mathrm{FP}+\mathrm{FN}},$$

(11) $$\mathrm{IoU}=\frac{\mathrm{TP}}{\mathrm{TP}+\mathrm{FP}+\mathrm{FN}},\;\mathrm{OA}=\frac{\mathrm{TP}+\mathrm{TN}}{\mathrm{TP}+\mathrm{TN}+\mathrm{FP}+\mathrm{FN}}.$$

## 4. Results

**Figure 3:**
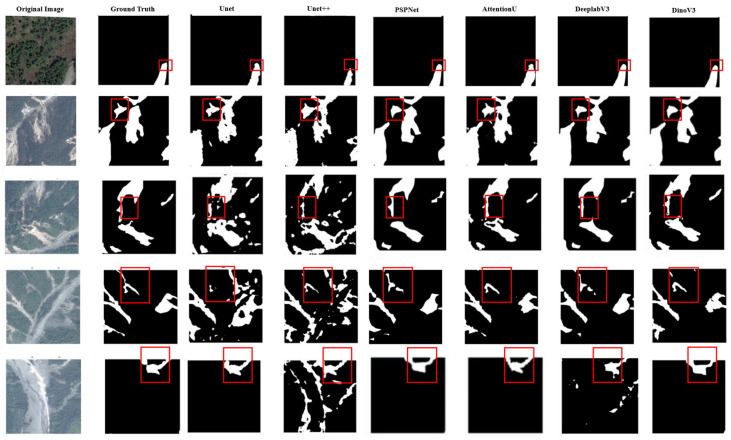
Comparison of segmentation results on the optical dataset. From left to right: original image, ground truth, U-Net, U-Net++, PSPNet, AttU-Net, DeepLabv3, and our proposed model. Red boxes indicate regions of interest for visual comparison.

To evaluate the efficacy of the proposed framework, we conducted a comprehensive comparative analysis against several established segmentation networks, including U-Net, U-Net++, PSPNet, AttU-Net, and DeepLabv3. These methods represent a diverse spectrum of encoder–decoder and multi-scale architectures, serving as robust baselines for semantic segmentation in remote sensing. The quantitative results on the Longxi optical dataset are summarized in Table 5, and qualitative comparisons are shown in Figure 3. Our model achieves the best performance across all metrics, with a Dice coefficient of 0.96 and an IoU of 0.938. Compared with the strongest baseline DeepLabv3, it shows improvements in both Precision and Recall, indicating fewer false positives while maintaining sensitivity to subtle landslide regions.

**Figure 4:**
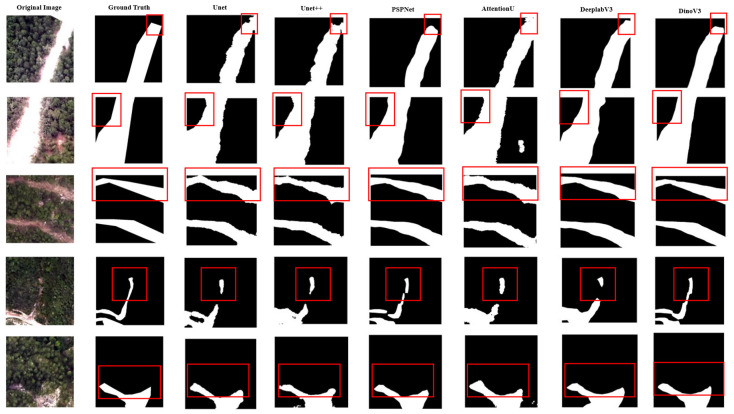
Comparison of segmentation results on the UAV dataset. From left to right: original image, ground truth, U-Net, U-Net++, PSPNet, AttU-Net, DeepLabv3, and our proposed model. Red boxes indicate regions of interest for visual comparison.

In addition, we evaluate the proposed framework on the Longxi UAV dataset following the same evaluation procedure and report both quantitative metrics and qualitative comparisons. Table 6 provides the quantitative results, and Figure 4 presents qualitative comparisons. From left to right, the columns correspond to the input image, the ground-truth mask, and the predictions produced by the baseline models and our proposed method. The baselines, such as U-Net and AttU-Net, can capture the coarse extent of landslides, but they often produce overly smooth boundaries and miss small structures. PSPNet may introduce false activations in background areas with similar textures. In contrast, our method generates masks that better match the ground truth, with clearer boundaries and reduced background noise.

## 5. Discussion

The results presented in Section 4 demonstrate that the proposed framework achieves higher segmentation accuracy than existing CNN-based baselines [33]. This performance is primarily attributed to the architectural characteristics of the model and the use of representations from multiple transformer layers.The transformer encoder can model global dependencies through self-attention, which helps capture the spatial structure of landslides that is difficult to represent with the local receptive fields of standard CNNs. On the Longxi satellite dataset, this leads to an IoU of 0.938 and a Precision of 0.961, indicating more accurate region delineation and fewer false activations in background areas with similar textures, such as bare soil or rocks. In addition, multi-level transformer representations provide complementary cues. Lower layers preserve boundary and texture information, while deeper layers encode highly level structure. Using information from multiple layers improves boundary delineation and the coverage of small landslide patches, contributing to a Dice of 0.960 and a Recall of 0.955 on the Longxi satellite dataset. Table 6 provides the results on the Longxi UAV dataset. The proposed method achieves a Dice of 0.965 and an IoU of 0.941, outperforming all baselines. The improved Precision and Recall indicate fewer false positives in background regions and better coverage of landslide areas in high-resolution UAV imagery. The multi-platform evaluation shows that the proposed framework achieves consistent improvements on both the satellite and UAV datasets. This suggests that the encoder does not rely on dataset-specific statistics but learns the essential structural features of landslides that generalize across sensors. Despite these strengths, limitations remain. Using representations from multiple layers increases memory consumption compared with lightweight CNNs. Moreover, the model still encounters difficulties in scenes with heavy shadows or extremely heterogeneous backgrounds. Future work will focus on adaptive feature selection to reduce redundancy, develop joint modeling and alignment strategies for combining satellite and UAV imagery, and integrate auxiliary topographic information such as DEM data to improve boundary definition in complex terrain.

## 6. Conclusions

This study presents a segmentation framework for landslide mapping from high-resolution remote sensing imagery. The proposed method uses a self-supervised DINOv3 encoder and a lightweight MLP decoder and leverages representations from multiple transformer layers to capture information from local textures with which to determine the broader geomorphological context. This design alleviates the limitations of local receptive fields in conventional CNNs and improves quantitative performance on the two Longxi datasets.

Experiments on the Longxi satellite and UAV datasets show consistent improvements over established baselines using the same evaluation protocol, with training conducted separately for each dataset. These results support the use of the proposed framework for practical landslide mapping across different acquisition platforms within the same study area.

Despite these advancements, challenges related to computational redundancy and performance in extreme lighting conditions remain. Future work will focus on optimizing the processing pipeline through adaptive feature selection and exploring multi-modal sensor fusion strategies, such as DEM data with optical imagery. Ultimately, this research underscores the potential of AI-driven representations to unify the analysis of remote sensing data, contributing to more resilient and automated geological hazard monitoring systems.

## Figures and Tables

**Table 1:** Typical landslide events in Jiuzhaigou Valley.

Occurrence Time	Trigger Factor	Landslide Scale (Area/Volume)	Direct Losses
8 August 2017	Earthquake (Jiuzhaigou $M_{s}$ 7.0 Earthquake)	Area $\approx 0.82\;{\mathrm{km}}^{2}$ Volume $\approx 2.1\times 10^{7}\;m^{3}$	176 houses damaged, 2573 people affected, ≈42km of roads cut off, 32 core scenic facilities damaged
17 August 2020	Heavy Rain (daily rainfall reaching 128 mm)	Area $\approx 0.15\;{\mathrm{km}}^{2}$ Volume $\approx 3.2\times 10^{5}\;m^{3}$	23 houses damaged, 312 people affected, ≈8km of roads cut off, 1 temporary barrier lake formed by blocked streams
29 July 2023	Heavy Rain (cumulative rainfall reaching 210 mm in 3 consecutive days)	Area $\approx 0.21\;{\mathrm{km}}^{2}$ Volume $\approx 5.8\times 10^{5}\;m^{3}$	41 houses damaged, 568 people affected, ≈15km of roads cut off, 2.3 km of scenic boardwalks damaged

**Table 2:** Comparison of different investigation methods.

Investigation Method	Advantages	Disadvantages
Ground Investigation	Intuitive data, enabling direct acquisition of surface feature information Low cost and flexible operation Real-time verification of judgments, conforming to on-site reality	Limited coverage and low survey efficiency Greatly restricted by terrain, difficult to conduct in dangerous and harsh areas Unable to detect the internal structure of the slope mass
Geological Exploration	Accurate detection of underground structures and sliding surface locations High data accuracy, providing direct basis for slope stability analysis Capable of revealing hidden information such as groundwater conditions	High cost, limiting large-scale application Low efficiency, belonging to point-line survey Some methods are destructive and may disturb the slope mass
Remote Sensing Technology	Wide coverage, suitable for large-area monitoring Strong terrain adaptability, capable of detecting remote areas Enabling long-term continuous dynamic monitoring	Data requires on-site verification and is prone to misjudgment Greatly interfered by weather and vegetation High cost for obtaining high-resolution data

**Table 3:** Overview of the datasets used in this study.

Dataset	Samples	Acquisition Time	Provider	Sensor Type	Resolution (m)	License
Longxi River (UAV)	2504	March 2011–May 2011	Sichuan Geomatics Center	UAV	0.5	Derivative Works License
Longxi River (SAT)	1769	March 2015–December 2015	China Centre for Resources Satellite Data and Application	GF-1 Satellite	0.5	Image License

**Table 4:** Summary of implementation details and hyperparameter settings.

Parameter	Value
Epochs	150
Batch size	32
Learning rate	$1\times 10^{-3}$
Weight decay	$5\times 10^{-5}$
Dropout	0.3
Optimizer	Adam
LR scheduler	CosineAnnealingLR
Gradient clipping	max_norm = 0.7
Early stopping	patience = 20
Patch size	256×256
GPU	NVIDIA A100 (80 GB)

**Table 5:** Comparison of segmentation performance on the Longxi optical image dataset.

Model	Dice	IoU	Precision	Recall	Accuracy
Unet [28]	0.935	0.905	0.934	0.930	0.927
Unet++ [29]	0.941	0.913	0.942	0.936	0.933
PSPNet [30]	0.944	0.917	0.945	0.939	0.936
AttU-Net [31]	0.938	0.909	0.937	0.933	0.931
DeepLabv3 [32]	0.948	0.924	0.949	0.944	0.939
Ours	0.960	0.938	0.961	0.955	0.972

**Table 6:** Comparison of segmentation performance on the Longxi UAV dataset.

Model	Dice	IoU	Precision	Recall	Accuracy
U-Net [28]	0.912	0.884	0.910	0.905	0.902
U-Net++ [29]	0.925	0.898	0.924	0.918	0.915
PSPNet [30]	0.931	0.905	0.933	0.927	0.924
AttU-Net [31]	0.940	0.916	0.942	0.936	0.932
DeepLabv3 [32]	0.954	0.930	0.956	0.948	0.962
Ours	0.965	0.941	0.964	0.958	0.975

## Data Availability

The data used in this paper are publicly available and can be accessed via the GitHub repository: https://github.com/Aizu0/CAS-Landslide-Dataset-production-code.git (accessed on 2 January 2026). The dataset is developed and maintained by the Artificial Intelligence Group at the Institute of Mountain Hazards and Environment, Chinese Academy of Sciences.
